# SpatialFinder: a human-in-the-loop vision-language framework for prioritizing high-value regions in spatial transcriptomics

**DOI:** 10.3389/fbinf.2026.1746714

**Published:** 2026-04-15

**Authors:** Jonathan Xu, Michelle Jiang, Shunsuke Koga, Nancy Zhang, Zhi Huang

**Affiliations:** 1 The Wharton School, University of Pennsylvania, Philadelphia, PA, United States; 2 College of Arts and Sciences, University of Pennsylvania, Philadelphia, PA, United States; 3 Hospital of the University of Pennsylvania, Philadelphia, PA, United States; 4 Department of Pathology and Laboratory Medicine, Perelman School of Medicine, University of Pennsylvania, Philadelphia, PA, United States

**Keywords:** clinical decision making, digital pathology, human-in-the-loop, spatial transcriptomics, vision-language models (VLMs)

## Abstract

Sequencing an entire spatial transcriptomics slide can cost thousands of dollars per assay, making routine use impractical. Focusing on smaller regions of interest (ROIs) based on adjacent H&E slides offers a practical alternative, but there is (i) no reliable way to identify the most informative areas from standard H&E images alone; and (ii) limited solutions for clinicians to prioritize the microenvironment of their own interests. Here we introduce SpatialFinder, a framework that combines a biomedical vision-language model (VLM) with a human-in-the-loop optimization pipeline to predict gene expression heterogeneity and rank high-value ROIs across routine H&E tissue slides. Evaluated across four Visium HD tissue types, SpatialFinder consistently outperforms VLM-only baselines for both diversity- and tumor-targeted ROI ranking, achieving Spearman’s ρ up to 0.89 and Overlap@10% up to 78.8%, an absolute 24.9 percentage-point gain over the strongest VLM. These results demonstrate the potential of human-AI collaboration to make spatial transcriptomics more cost-effective and clinically actionable.

## 1 Introduction

Spatial transcriptomics (ST) enables *in situ* quantification of gene expression across intact tissue sections, preserving spatial context to map transcriptional activity within anatomical niches (Ståhl et al., 2016). This spatial resolution makes it possible to distinguish expression patterns in specific regions, such as a tumor’s core versus its invasive edge, revealing interactions that bulk or single-cell RNA-seq would miss (Arora et al., 2023). Modern ST technologies enable gene expression profiling across entire tissue sections (Williams et al., 2022), but practical adoption is hindered by high cost and scalability challenges (Smith et al., 2024). Sequencing a single slide can cost up to $15,000 using advanced platforms like 10xGenomics Visium HD (Center for Cellular Profiling, Brigham and Women’s Hospital, 2025; Pitt Center for Advanced Genomics, University of Pittsburgh, 2025). These assays remain too expensive and thus impractical for usage in widespread research or clinical settings (Zhu et al., 2025; Piñeiro et al., 2022). Moreover, large portions of a tissue slide may offer little informative value or repeated patterns, leading to significant investment in sequencing regions that contribute minimal biological insight (Ni et al., 2022; Jones et al., 2024).

**FIGURE 1:**
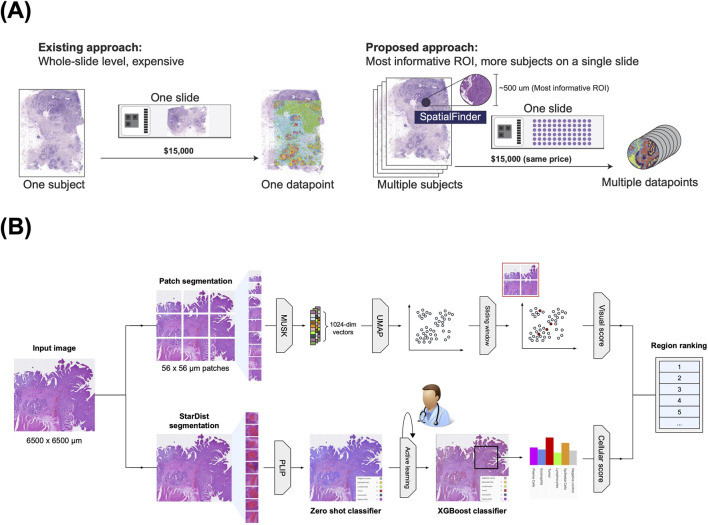
**(A)** Current spatial omics cost upwards of $15,000 per slide, creating a high cost bottleneck for broader application. Our proposed alternative addresses this by identifying only the most relevant regions within each slide for gene sequencing, dramatically increasing the number of subjects that can be analyzed at the same cost. **(B)** An overview of our approach. SpatialFinder utilizes a sliding-window procedure on the input image to extract adjacent patches for analysis. Image patches are encoded into high-dimensional feature vectors, which are then projected into a lower-dimensional space via UMAP for visualization and evaluation. In parallel, we train a nuclei classifier through an active-learning loop with expert pathologist input to classify cells accurately from H&E. Regions are ranked by the weighted scores from both arms of the proposed pipeline.

This inefficiency motivates the need for methods to intelligently *target* sequencing to the most informative subregions of a tissue sample (Cui et al., 2024). By reliably identifying smaller areas that reflect the tissue’s gene expression diversity, we could focus sequencing efforts where they matter most, drastically reducing costs while preserving essential molecular information (Qian et al., 2025). Such an approach would make spatial transcriptomics much more accessible for both research and clinical use in the future (see Figure 1A).

### 1.1 Related work

In the past decade, artificial intelligence (AI) has made rapid strides in analyzing whole-slide histology images (McGenity et al., 2024). At the forefront of this progress are vision-language models (VLMs), which learn joint representations of images and text (Radford et al., 2021). In pathology, VLMs can localize regions matching natural language prompts, such as “tumor region with dense lymphocyte infiltration”, even without explicit training on those patterns (Stegeman et al., 2024; Rahaman et al., 2025). By aligning semantic concepts with visual features, VLMs offer a flexible way to detect novel or rare tissue patterns that may reflect underlying molecular or clinical features (Chen et al., 2025; Vaidya et al., 2025).

Recent efforts in both bioinformatics and AI have explored strategies to reduce the experimental burden of spatial transcriptomics (Chelebian et al., 2025). Image-to-omics prediction models have been developed to computationally infer spatial gene expression patterns directly from histology images (Nonchev et al., 2025). For example, convolutional neural networks and graph neural networks have been applied to predict transcriptomic profiles in unseen tissue regions, offering a promising *in silico* alternative to exhaustive sequencing (Wang et al., 2025). However, a limitation of purely computational approaches is the uncertainty in predictions, which still benefits from validation via actual sequencing in critical regions (Sun et al., 2024). Other related work has focused on identifying regions-of-interest in pathology slides using weakly supervised learning and anomaly detection, to guide pathologists or downstream analyses (Nan et al., 2025; Dippel et al., 2024).

While these data-driven AI models are increasingly powerful, they often act as black boxes, lacking transparency and sometimes producing clinically implausible results (Kiseleva et al., 2022; Muhammad and Bendechache, 2024). Most importantly, expert clinicians have limited access to steer or evolve medical AI systems in line with clinical and research needs (Banerji et al., 2025). To keep AI tools accurate and trustworthy in medicine, especially in the scenario of choosing ROI for reducing ST costs, a human-in-the-loop component is essential: integrating expert feedback throughout training and deployment can align AI models’ predictions with clinicians’ expectations (Sezgin, 2023). To let clinicians be part of this process, active learning is one effective strategy, where the model selectively queries experts to label uncertain or informative samples and retrains accordingly (Kim et al., 2024). This process focuses the model on clinically relevant features with far fewer annotations than traditional methods (Zhou et al., 2021). Studies have shown that such human-guided pipelines not only improve performance but also reduce labeling effort and training time (Sadafi et al., 2023; Patil et al., 2023).

Unlike previous approaches, our proposed SpatialFinder identifies optimal ROIs for spatial transcriptomics by leveraging vision-language features to capture semantic heterogeneity. Uniquely, it incorporates a human feedback loop to iteratively refine predictions. To our knowledge, this is the first approach to integrate a foundation vision-language model (VLM) with expert pathologist input for region selection in this context. Furthermore, by benchmarking selected subregions against ground truth spatial omics data, we introduce an evaluation framework for region selection, a gap not explicitly addressed in existing studies. In evaluations across four Visium HD tissue types, SpatialFinder demonstrated up to 89% agreement with ground truth region rankings and achieved substantially greater spatial coverage of top regions compared to leading baselines. Notably, just 5–10 min of expert feedback yields up to a 50 percentage-point improvement in overlap with ground truth among the highest-ranked regions, underscoring the practical impact of human-in-the-loop refinement.

## 2 Materials and methods

We present SpatialFinder, a framework that combines a vision-language model with human-in-the-loop supervision to identify informative subregions in histology images for spatial transcriptomics (see Figure 1B). The method consists of two parallel components: (i) a pathologist-guided cellular classifier trained on hematoxylin and eosin (H&E) stained slides for fine-grained, single-cell recognition of histological patterns, and (ii) a VLM that encodes image patches into high-dimensional embeddings for zero-shot interpretation of complex tissue structures. By combining expert-labeled cellular features with the semantic generalization of a VLM, our system robustly identifies regions likely to capture diverse and relevant gene expression profiles.

### 2.1 Data curation and preprocessing

We curated a dataset of high-resolution Visium HD H&E-stained whole-slide images (WSIs) from 10xGenomics, spanning four tissue types: lung cancer (10x Genomics, 2024a), colon cancer (10x Genomics, 2024b), non-cancerous kidney (10x Genomics, 2025a), and prostate cancer (10x Genomics, 2025b). Each dataset package includes the full-resolution tissue image in TIFF format, filtered feature-barcode matrices in HDF5 format, spatial coordinate files (tissue_positions.parquet), and scaling factor metadata (scalefactors_json.json).

We first perform automated cropping to isolate the tissue capture region from the full WSI. Crop boundaries are determined by identifying the minimum bounding rectangle encompassing all tissue spots marked in the tissue_positions.parquet file, using pixel coordinates from the pxl_col_in_fullres and pxl_row_in_fullres columns. We add padding equal to the spot diameter (obtained from scalefactors_json.json) to ensure complete capture of spot boundaries. The cropped region preserves original image metadata including magnification and resolution specifications.

After cropping, we perform comprehensive cell nucleus segmentation on each tissue region. We apply the pre-trained StarDist deep learning model (2D_versatile_he, configured for H&E-stained tissue) without additional fine-tuning to detect and delineate each cell nucleus in the image (Schmidt et al., 2018). The model processes each cropped region in overlapping tiles of 4096 × 4096 pixels with 256-pixel overlap, using a probability threshold of 0.3 and non-maximum suppression threshold of 0.3 for nuclei detection. This yields a detailed segmentation mask marking the precise location and boundaries of each nucleus in the tissue, with each nucleus represented by contour polygons and centroid coordinates stored alongside detection probability scores in HDF5 format. Following segmentation, we extract 224 × 224 pixel patches centered on each nucleus centroid and compute image embeddings using the pre-trained Pathology Language-Image Pretraining (PLIP) model (vinid/plip; ViT-B/32 architecture; 512-dimensional embeddings) (Huang et al., 2023). PLIP is used as a frozen feature extractor without additional fine-tuning. We use PLIP for these nucleus-centered patches for two reasons. First, PLIP’s joint image-text embedding space enables rapid zero-shot initialization of cell labels via text prompts (e.g., “tumor cell,” “immune cell”), which is then refined through active learning. Second, PLIP’s ViT-B/32 architecture processes 49 tokens per image (patch size 32 × 32), compared to 196 tokens for CONCH’s ViT-B/16 (patch size 16 × 16), yielding substantially faster inference. This speed advantage is critical for nucleus-level embeddings because a pathologist is waiting to begin annotation once segmentation and embedding are complete; minimizing this preprocessing time enables rapid initiation of the human-in-the-loop workflow. These embeddings, which encode morphological features of each nucleus, are stored in the HDF5 file under SegmentationNode/embedding for use in subsequent classification steps. The resulting segmentation and pre-computed embeddings serve as the basis for subsequent pathologist-guided cell type annotation and classification.

### 2.2 Morphological feature extraction with VLM

To capture high-level morphological features for ROI ranking, we require patch-level embeddings that represent broader tissue architecture and semantic heterogeneity beyond individual nuclei. Each cropped tissue image is divided into a non-overlapping grid of small patches (224 × 224 pixels each), with incomplete edge tiles padded with white pixels to maintain uniform patch dimensions. Patches are stored in a compressed Zarr array format for efficient batch processing. During patch extraction, we apply an initial filter to exclude patches that are completely white (all pixel values equal to 255) or completely black (all pixel values equal to 0), removing empty background regions before model processing.

**FIGURE 2:**
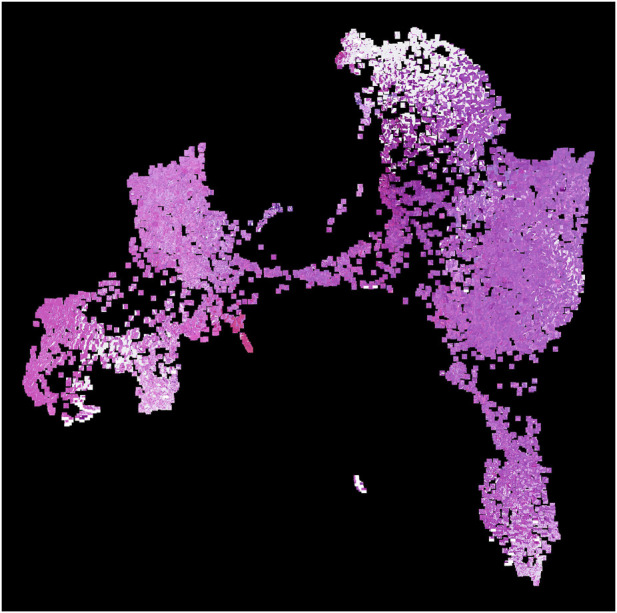
Two-dimensional UMAP projection of patch-level embeddings extracted from our colon whole-slide image. Each point represents an image patch with the corresponding patch thumbnail overlaid. The visualization reveals coherent spatial and morphological clusters within the tissue.

The remaining patches are processed with the Multimodal transformer with Unified maSKed modeling (MUSK), a transformer-based VLM pre-trained on large-scale pathology image-text data (Xiang et al., 2025). We selected MUSK over alternative pathology VLMs (PLIP, CONCH) for two reasons. First, MUSK was trained on a substantially larger and more diverse corpus: 50 million pathology images and 1 billion text tokens, compared to 208K image-text pairs for PLIP (Huang et al., 2023) and 1.17 million for CONCH (Lu et al., 2024a). We hypothesize this broader pretraining yields more generalizable morphological representations across tissue types. Second, MUSK produces 1024-dimensional embeddings (vs. 512 for PLIP and CONCH), providing a richer feature space for capturing subtle morphological variation in our UMAP-based diversity scoring. Unlike nucleus-level embeddings, patch-level embeddings can be computed offline without a pathologist waiting, so we prioritize representation quality over inference speed. MUSK is used as a frozen feature extractor without additional fine-tuning. Each 224 × 224 patch is resized to 384 × 384 pixels and center-cropped to match MUSK’s input requirements, then normalized using ImageNet Inception statistics (mean = [0.485, 0.456, 0.406], std = [0.229, 0.224, 0.225]). The model architecture (musk_large_patch16_384) processes patches in batches of 4 and generates a 1024-dimensional embedding per patch, encoding rich morphological semantics for zero-shot inference (see Figure 2 for a 2D UMAP of the embeddings). Patches are processed sequentially through the model using GPU acceleration, with embeddings stored alongside their spatial coordinates.

To ensure feature quality, we apply a two-stage filtering approach post-embedding. The first stage (Tier 1) identifies and removes strong background patches using a combination of mean grayscale intensity (threshold ≥ 235) and low variance (standard deviation \lt 20). When enabled, an additional color check verifies that at least 85% of pixels have all RGB channels ≥ 230, confirming uniform bright background. The second stage (Tier 2) removes remaining low-variance tissue patches that may lack informative morphological content, using a standard deviation threshold of \lt 10. This two-tier filtering approach ensures that only patches with meaningful morphological variation are retained for downstream analysis, improving the signal-to-noise ratio of the morphological feature space. We further verified that this background filtering is not sensitive to small changes in the intensity and variance thresholds: across tissues, the fraction of sampled patches flagged as background remained nearly unchanged when varying the mean-intensity threshold from 230–240 and the standard-deviation threshold from 15–25 (Supplementary Table S6).

### 2.3 Pathologist classification via active learning

To achieve fine-grained cellular classification, we employ a hybrid active learning workflow powered by NuClass, a custom classification tool integrated within our in-house TissueLab software platform (Li et al., 2025). This workflow begins with an automated, zero-shot labeling of all segmented nuclei. The classification process reads pre-computed PLIP image embeddings from SegmentationNode/embedding in the HDF5 file. For zero-shot classification, we use the text encoder from the same PLIP model to embed pathologist-defined text labels (e.g., “tumor cell,” “immune cell”) as feature vectors. Nuclei are assigned the label with the highest cosine similarity between their pre-computed image embedding and the text label embeddings, enabling rapid, semantic classification without manual annotation. This zero-shot approach leverages the shared embedding space learned by PLIP during pre-training on large-scale pathology image-text pairs, where semantically similar concepts are mapped to nearby points in the embedding space.

This initial, AI-generated map serves as a starting point for an expert pathologist, who uses TissueLab’s interactive interface to label small, representative subsets of nuclei. The pathologist can navigate the tissue spatially, identify misclassified or uncertain regions, and provide ground truth labels for selected nuclei or nuclei groups. These expert annotations, comprising each cell’s pre-computed image embedding (from SegmentationNode/embedding) and its correct label, form a training set for a supervised XGBoost classifier (Chen and Guestrin, 2016). We use XGBoost with default hyperparameters (100 estimators, max_depth = 6, learning_rate = 0.3, objective = multi:softprob); no custom tuning was performed, as the classifier generalizes well across tissues with the pre-trained PLIP embeddings as input. The XGBoost model learns to map the high-dimensional PLIP embeddings to cell type labels, capturing non-linear decision boundaries that improve upon the linear cosine similarity approach used in zero-shot classification.

Once trained on this curated dataset, the XGBoost classifier is reapplied to predict labels for all nuclei across the tissue. The pathologist can then iteratively refine the results by providing additional labels, particularly in areas of uncertainty or where the model predictions disagree with visual inspection. With each cycle, the XGBoost classifier is retrained on the cumulative set of annotations, rapidly improving its accuracy and converging into a robust model with only a few hundred expert labels. This iterative active learning process efficiently combines large-scale automated analysis with precise expert knowledge, producing a spatially-resolved cell type map for hundreds of thousands of cells. The final classification results are stored alongside spatial coordinates and segmentation boundaries (please see Supplementary Data), enabling downstream spatial analysis of cell type distributions and tissue architecture.

**FIGURE 3:**
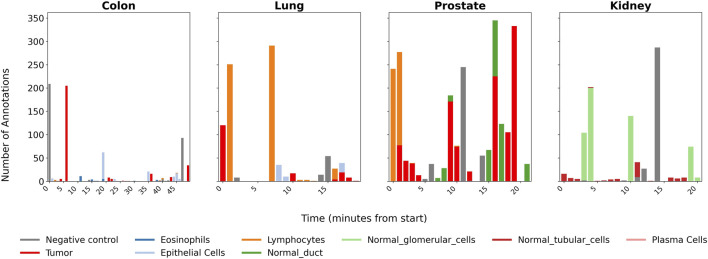
Distribution of annotation timestamps for the four Visium HD tissue types used to train the active-learning classifiers. Each bar represents the number of nuclei annotated for a given cell or tissue category at a specific time point during the session. Across tissues, 756–2,282 nuclei were labeled over 31–48 annotation cycles (mean 16–49 nuclei per cycle), with total session durations of 19–49 min (mean ≈ 27 min). Annotations were performed via rectangle selection (85%–100% of labels) or polygon selection for irregular regions. The temporal distribution illustrates the pathologist’s iterative refinement strategy, alternating between cell types and tissue regions to rapidly build a representative training set.

Across the four Visium HD tissues used in this study, segmentation yielded 154,648–225,139 nuclei per slide. A single pathologist annotated 756–2,282 nuclei per tissue (0.4%–1.0% of total nuclei) over 31–48 annotation cycles, with each cycle corresponding to a rectangle or polygon selection labeling multiple nuclei simultaneously (mean 16–49 nuclei per cycle, range 1–333). The majority of annotations (85%–100%) were made via rectangle selection, with polygon selection used for irregularly shaped regions in colon and kidney tissues. Total annotation time ranged from 19–49 min per tissue (mean ≈ 27 min). Cell type categories ranged from 3 (kidney: tubular, glomerular, negative control) to 6 (colon: tumor, epithelial, lymphocytes, eosinophils, plasma cells, negative control). Within each tissue, certain cell types dominated the annotation effort: tumor cells comprised 48% of prostate annotations, lymphocytes 65% of lung annotations, negative control regions 41% of colon annotations, and glomerular cells 55% of kidney annotations, reflecting the pathologist’s strategy of focusing on the most morphologically distinctive or clinically relevant populations. Figure 3 shows the temporal distribution of annotations across cell types for each tissue.

### 2.4 Region of interest generation and scoring

To prioritize regions of interest (ROIs) for spatial transcriptomics sequencing, we employ a hybrid scoring system that integrates VLM-based visual features with pathologist-annotated cell type maps. We systematically scan the cropped tissue image using an overlapping sliding window approach with a step size of one patch, generating a comprehensive grid of candidate ROIs for evaluation. We set the default stride to one patch to maximize spatial coverage and reduce grid-alignment artifacts when selecting a small number of ROIs for sequencing; we then apply a non-overlapping selection step to ensure the final chosen ROIs do not overlap. To assess whether overlap inflates evaluation metrics, we additionally evaluated larger strides (2 and 4 patches) and found that Spearman’s ρ remains stable across strides, indicating that our choice of stride does not inflate local correlations during evaluation (Supplementary Table S2).

#### 2.4.1 Sliding window ROI generation

Each ROI is defined as an 8×8 patch window, corresponding to 1792×1792 pixels ( 8×224 pixels per patch) at the original image resolution. We selected this window size to align with the field of view of spatial transcriptomics platforms: at our imaging resolution (∼0.274 μ m/pixel from scalefactors_json.json), each 224-pixel patch corresponds to ∼61 μ m, such that an 8×8 window spans approximately 491×491 μ m. This closely matches the ∼500 × 500 μ m field of view of spatial transcriptomics devices such as the CosMX Spatial Molecular Imager, ensuring that predicted ROIs are directly compatible with downstream sequencing workflows. This window size represents approximately 0.6% of the total 6.5×6.5 mm Visium HD capture area. The sliding window scans the entire patch grid with a step size of one patch, generating approximately 10,000 overlapping ROIs per slide. For each ROI, we compute two distinct scores: a Regional Diversity Score for unsupervised discovery of biological complexity, and a Targeted Conditional Score for hypothesis-driven searches.

#### 2.4.2 Regional Diversity score

The Regional Diversity Score quantifies biological heterogeneity within each ROI by combining visual and cellular diversity measures.

Visual Diversity Component: We compute morphological diversity using the MUSK patch embeddings generated during the feature extraction step. All 1024-dimensional patch embeddings are first reduced to 30 dimensions using UMAP (McInnes et al., 2018) with parameters n_neighbors=15 , min_dist=0.1 , and random_state=42 to ensure reproducibility. The UMAP reduction is fit globally on all patches, then each ROI’s visual diversity score is computed as the median pairwise Euclidean distance between the 30-dimensional embeddings of patches within that ROI. This metric captures morphological variation: higher scores indicate regions with diverse tissue architecture (e.g., mixed glandular, stromal, and tumor areas), while lower scores indicate morphologically uniform regions. We additionally evaluated sensitivity to UMAP dimensionality in ablations (Supplementary Material).

Cellular Diversity Component: We compute compositional diversity using the pathologist-annotated cell type map. For each ROI, we count the number of nuclei belonging to each cell type across all patches within the window. We then compute Shannon entropy (Shannon, 1948) (base 2) of the resulting cell type distribution: $H=-\sum_{i}p_{i}\log_{2}\left(p_{i}\right)$ , where $p_{i}$ is the proportion of cells belonging to cell type i within the ROI. To account for cell density, we multiply the entropy by log(1+N) , where N is the total number of cells in the ROI, giving higher scores to regions with both high diversity and sufficient cell density. ROIs with fewer than 10 cells are assigned a score of zero.

Score Combination: Before combination, both visual and cellular diversity scores are normalized to the range [0, 1] using min-max normalization across all ROIs. The final Regional Diversity Score is computed by Equation 1:

$$S_{\text{Diversity}}=w\cdot {\text{Score}}_{\text{visual}}+\left(1-w\right)\cdot {\text{Score}}_{\text{cellular}}$$ (1)

where w is a tunable weight parameter (default 0.5) controlling the relative contribution of visual versus cellular diversity.

#### 2.4.3 Targeted Conditional Score

The Targeted Conditional Score identifies ROIs matching a user-defined biological query by combining text-guided VLM similarity with target cell abundance from the classifier.

Text-Guided VLM Similarity Component: For hypothesis-driven searches, users provide a text prompt describing the biological phenotype of interest (e.g., “tumor,” “immune infiltration,” “stromal reaction”). The text prompt is embedded using the same MUSK model’s text encoder to generate a 1024-dimensional text embedding. We then compute cosine similarity between this text embedding and each patch’s pre-computed MUSK image embedding within the ROI. To aggregate patch-level similarities into an ROI-level score, we use the 90th percentile (quantile 0.9) of cosine similarity scores across all patches in the window, which emphasizes strong matches while reducing sensitivity to outliers. To further enhance contrast and emphasize regions with high similarity, we apply an exponential transformation by raising the aggregated score to a power (default: exponent =10.0 ), effectively amplifying differences between moderately and highly similar regions. We report ablations over these aggregation hyperparameters in Supplementary Material.

Cell Abundance Component: The cellular component quantifies the abundance of a specific target cell type within the ROI. We count the number of nuclei belonging to the target cell type (e.g., tumor cells) across all patches within the 8×8 window, then apply a log-transformation: log(1+count) to handle the wide dynamic range of cell counts while maintaining sensitivity to low-abundance regions.

Score Combination: Similar to the diversity score, both components are normalized to [0, 1] using min-max normalization before combination. The final targeted conditional score is computed by Equation 2:

$$S_{\text{Targeted}}=w\cdot {\text{Score}}_{\text{VLM}}+\left(1-w\right)\cdot {\text{Score}}_{\text{count}}$$ (2)

where w is a tunable weight parameter (default 0.5) controlling the relative contribution of text-guided similarity versus cell abundance.

#### 2.4.4 ROI ranking and selection

**FIGURE 4:**
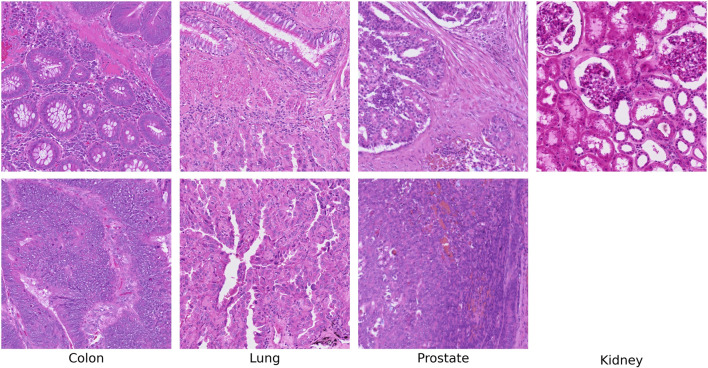
Top row: regions of interest ranked highest by predicted cellular diversity. Bottom row: regions ranked highest when conditioned on tumor presence. The kidney sample is non-cancerous and therefore does not include a tumor-conditioned entry.

Each scoring metric (Regional Diversity and Targeted Conditional) independently ranks all ROIs by their respective scores. Top regions are selected using a greedy non-overlapping algorithm that processes ROIs in descending score order: each ROI is selected only if its center is separated from all previously selected ROI centers by at least the window size in both row and column dimensions. This ensures selected ROIs do not overlap spatially and promotes diverse spatial sampling across the tissue. The final ranked lists, along with spatial coordinates and visualization images, are generated for downstream sequencing prioritization. The top-ranked regions for each scoring method are highlighted in Figure 4.

### 2.5 Experimental validation

To validate our ROI ranking methodology, we benchmarked model predictions against ground truth rankings derived from Visium HD spatial transcriptomics data. The Visium HD platform natively provides gene expression measurements at a 2×2 μ m pixel resolution. For analysis, we aggregate these native pixels into 8×8 μ m bins, or “spots,” which correspond roughly to near single-cell areas. We adopt this aggregated 8×8 μ m resolution to reduce sub-cellular noise and mitigate expression spillover between adjacent pixels. We constructed ground truth rankings by classifying each binned spot based on marker gene expression, then aggregating spot-level classifications into regional scores using the same 8×8 patch sliding-window approach employed by our model.

#### 2.5.1 Ground truth spot classification

We defined cell type-specific marker gene sets based on canonical markers curated from biological literature and prior databases (Dalerba et al., 2011; He et al., 2021; Subramanian et al., 2019; Gokey et al., 2021; Travaglini et al., 2020; Song et al., 2022; Agarwal et al., 2015; Hu et al., 2023). A full list of marker genes is available in our Supplementary Data. Marker selection was tailored to each tissue type:

- Lung and colon cancer: Tumor cells were identified using epithelial and carcinoma markers such as *EPCAM* and *CEACAM5.* Immune cell types were defined by *PTPRC* (pan-immune), *CD3D* and *CD8A* (T cells), and *MS4A1* (B cells). Stromal and endothelial populations were marked by *COL1A1* and *PECAM1*, respectively.
- Prostate cancer: Tumor cells were labeled using *AMACR* and *KLK3* (encoding *PSA*). Basal and luminal epithelial cells were identified with cytokeratins like *KRT5* and *KRT8.*
- Non-cancerous kidney tissue: Cell types were annotated by functional nephron markers—*NPHS1* (podocytes), *LRP2* (proximal tubule), and *UMOD* (loop of Henle).

For each spot, we computed a cell type-specific expression score by taking the mean normalized expression across all marker genes within each marker set. Prior to scoring, gene expression counts were normalized to 10,000 total counts per spot (accounting for library size variation), then log-transformed using log(1+x) to stabilize variance. Each spot was assigned to the cell type with the highest expression score, provided that score exceeded a minimum threshold of 1.0 normalized expression units; spots failing to meet this threshold were labeled as “Unclassified.” This classification procedure yielded a spatially-resolved cell type map at the resolution of individual Visium HD spots.

#### 2.5.2 Ground truth regional ranking

To construct regional ground truth rankings, we applied an 8×8 patch sliding window (matching our model’s inference window) across the entire patch grid. For each windowed region, we aggregated the cell type classifications of all Visium HD spots spatially contained within that region.

For diversity-based ground truth, we computed the Shannon entropy (base 2) of the spot-level cell type distribution within each window: $H=-\sum_{i}p_{i}\log_{2}\left(p_{i}\right)$ , where $p_{i}$ is the proportion of spots belonging to cell type i within the ROI. To account for spot density, we multiplied the entropy by log(1+N) , where N is the total number of spots in the region, rewarding regions with both high diversity and sufficient spot coverage. Regions containing fewer than 10 spots were assigned a score of zero, ensuring robust statistical estimates. The resulting scores were ranked in descending order to produce a ground truth diversity ranking.

For tumor-targeted ground truth, we scored each region based on the total count of spots classified as tumor within the window. Regions were ranked by this count in descending order, prioritizing tumor-rich regions.

#### 2.5.3 Evaluation metrics

We evaluated our model’s ROI rankings against these ground truth measures using three complementary metrics:

- Spearman’s rank correlation (ρ) : Measures global concordance between model and ground truth rankings, indicating overall prioritization accuracy (Spearman, 2010). The correlation is computed over the set of regions common to both rankings, with ranks assigned based on position in each ordered list. A value of ρ=1.0 indicates perfect agreement, while ρ=0 indicates no monotonic relationship.
- Top-K overlap (Overlap@K): Computes the percentage overlap between the top K regions identified by the model and by the ground truth, highlighting performance at the high-priority end of the list. Specifically, for a given K, we compute: $\text{Overlap@K}=\frac{\left|{\text{Top}-\text{K}}_{\text{model}}\cap {\text{Top}-\text{K}}_{\text{GT}}\right|}{K}\times 100\%$ , where regions are considered matched if their coordinates are identical. This metric emphasizes the model’s ability to identify the most biologically relevant regions, which is critical for downstream sequencing prioritization.
- Intersection over Union (IoU@K): Assesses spatial alignment by measuring the area-based overlap between the top K predicted and ground truth regions (Jaccard, 1912). We compute Set IoU@K, which treats the top K regions as a combined set of patches and computes the overall intersection-over-union ratio. This metric captures the overall spatial agreement of the entire top-K set, accounting for the 8×8 patch window size when computing bounding box overlaps.

These metrics provide complementary perspectives: Spearman’s correlation captures global ranking agreement, Top-K overlap measures exact identification of high-priority regions, and Set IoU quantifies spatial precision. Together, they comprehensively assess both the ranking accuracy and spatial localization performance of our ROI selection methodology.

## 3 Results

We assessed our hybrid model on four distinct Visium HD tissue types, evaluating both cell diversity and tumor presence. Across the board, our model consistently outperformed baseline methods in identifying biologically meaningful regions of interest.

### 3.1 SpatialFinder model surpasses VLM baselines

To establish a quantitative reference point, we benchmarked SpatialFinder against three prominent vision-language models: PLIP and CONCH—state-of-the-art VLMs in pathology—and MUSK, the vision-language component of our own hybrid system.

All models were evaluated using an identical preprocessing and inference pipeline, including tissue cropping, 224×224 patching, background filtering, UMAP-based dimensionality reduction, and an 8×8 sliding window for scoring regions of interest (ROIs). The critical distinction is that baseline models do not incorporate human-in-the-loop cellular insights: they exclude nuclei segmentation and pathologist-informed cell classification. For the diversity task, baseline scoring relied solely on the UMAP spread of patch embeddings (w=1) , while the targeted task used only text-to-image similarity (w=1) , computed via the 90th-percentile cosine similarity between patch embeddings and the text prompt “tumor.”

PLIP and CONCH are used strictly zero-shot as plug-in encoders, while MUSK (VLM-only) serves as an internal ablation to isolate the added value of the classifier. As a sanity check, we also evaluated random ROI selection (Supplementary Table S5): random selection achieved Spearman’s ρ=0 (no correlation) and Overlap@10% ≈ 10%, the expected chance level when randomly sampling 10% of regions. In contrast, SpatialFinder achieves Overlap@10% of 25%–78%, representing a 2.5× to 7.8× improvement over random chance.

As detailed in Table 1, 2, SpatialFinder matched or outperformed baselines across all tissue types for both diversity and tumor detection tasks, and we report Spearman’s ρ with 95% bootstrap confidence intervals for each tissue and model. On the tumor benchmark, SpatialFinder achieved Spearman’s ρ=0.89 in lung tissue and 78.8% Overlap@10% in prostate tissue; on the diversity benchmark, it reached ρ=0.80 in kidney tissue.

**TABLE 1:** Performance of SpatialFinder against other baseline VLMs across four datasets on the cell diversity benchmark. Bold texts indicate the best-performing result. SpatialFinder results report the best-performing weight from a sweep over w∈{0.0,0.2,0.4,0.6,0.8,1.0} for each tissue; VLM-only baselines use visual features only. We report Spearman’s ρ with 95% bootstrap confidence intervals computed by resampling the set of evaluated windows with replacement (1,000 bootstrap samples) within each tissue.

Tissue	Model	ρ [95% CI]	Overlap@10%	IoU@10%
Prostate	PLIP	0.484 [0.467, 0.501]	2.92%	0.169
	CONCH	0.434 [0.417, 0.450]	1.23%	0.147
	MUSK	0.650 [0.637, 0.662]	38.68%	0.478
	SpatialFinder	**0.762 [0.753, 0.770]**	**47.45%**	**0.559**
Lung	PLIP	0.458 [0.442, 0.475]	8.82%	0.249
	CONCH	0.521 [0.505, 0.537]	12.19%	0.269
	MUSK	0.594 [0.579, 0.609]	17.24%	0.366
	SpatialFinder	**0.653 [0.640, 0.668]**	**25.27%**	**0.406**
Colon	PLIP	0.167 [0.147, 0.188]	13.20%	0.172
	CONCH	0.161 [0.140, 0.184]	23.70%	0.249
	MUSK	0.196 [0.173, 0.215]	31.90%	0.324
	SpatialFinder	**0.365 [0.345, 0.382]**	**38.10%**	**0.445**
Kidney	PLIP	0.660 [0.645, 0.673]	32.26%	0.445
	CONCH	0.639 [0.624, 0.653]	33.77%	0.482
	MUSK	0.701 [0.689, 0.711]	37.36%	0.540
	SpatialFinder	**0.801 [0.794, 0.809]**	**48.30%**	**0.594**

**TABLE 2:** Performance of SpatialFinder against other baseline VLMs across three datasets on the tumor benchmark. Bold texts indicate the best-performing result; the Visium HD kidney sample is non-cancerous and therefore excluded from tumor evaluations. SpatialFinder results report the best-performing weight from a sweep over w∈{0.0,0.2,0.4,0.6,0.8,1.0} for each tissue; VLM-only baselines use text similarity only. We report Spearman’s ρ with 95% bootstrap confidence intervals computed by resampling the set of evaluated windows with replacement (1,000 bootstrap samples) within each tissue.

Tissue	Model	ρ [95% CI]	Overlap@10%	IoU@10%
Prostate	PLIP	0.222 [0.201, 0.241]	23.49%	0.278
	CONCH	0.829 [0.822, 0.835]	44.15%	0.486
	MUSK	0.833 [0.827, 0.838]	53.87%	0.553
	SpatialFinder	**0.857 [0.849, 0.865]**	**78.77%**	**0.775**
Lung	PLIP	0.254 [0.233, 0.272]	0.00%	0.062
	CONCH	0.826 [0.819, 0.833]	35.08%	**0.492**
	MUSK	0.653 [0.640, 0.667]	1.98%	0.179
	SpatialFinder	**0.886 [0.882, 0.890]**	**37.26%**	0.401
Colon	PLIP	0.352 [0.336, 0.369]	11.30%	0.217
	CONCH	0.619 [0.606, 0.632]	15.70%	0.309
	MUSK	0.527 [0.511, 0.544]	11.70%	0.243
	SpatialFinder	**0.623 [0.609, 0.637]**	**19.10%**	**0.355**

SpatialFinder’s robust performance across lung, colon, prostate, and kidney datasets underscores its versatility. Notably, CONCH outperformed SpatialFinder on the lung tumor IoU@10% metric (0.49 vs. 0.40), though SpatialFinder still achieved higher Spearman’s ρ (0.89 vs. 0.83) and comparable Overlap@10% (37.3% vs. 35.1%) on the same benchmark. This discrepancy highlights an important distinction between metric types: IoU measures spatial contiguity of selected regions, whereas ρ and Overlap assess ranking fidelity and set-level agreement. CONCH’s advantage in IoU suggests that its purely visual embeddings may capture spatially coherent tumor boundaries more effectively, while SpatialFinder’s cellular scoring, which aggregates predictions across individual nuclei, can fragment contiguous regions when cell-type predictions are heterogeneous at the margin.

This pattern was unique to lung tissue; across all other tissues and benchmarks, SpatialFinder matched or exceeded CONCH on all three metrics. We hypothesize that lung’s distinct morphology, characterized by abundant alveolar space and sparse cellularity, may reduce the discriminative power of nucleus-level features relative to patch-level visual embeddings. For tissues with denser cellular architecture (e.g., prostate glands, colonic crypts), the nucleus-level classifier provides substantial gains, as evidenced by SpatialFinder’s 29 percentage-point IoU advantage over CONCH in prostate tumor detection.

These findings suggest that VLM-only approaches like CONCH may be preferable when spatial contiguity is paramount and tissue morphology is sparse, whereas hybrid approaches like SpatialFinder offer more robust performance across diverse tissue types and evaluation criteria. The complementary strengths of each approach underscore the value of reporting multiple metrics and considering tissue-specific characteristics when selecting ROI prioritization strategies.

### 3.2 Consistent performance across Top-K regions

**FIGURE 5:**
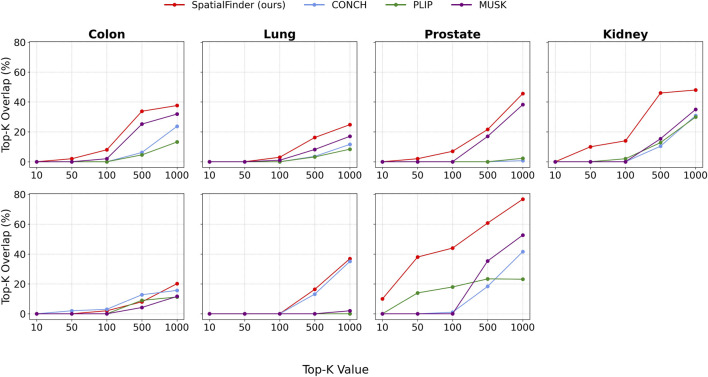
Top-K selection accuracy for each tissue type, evaluated across multiple K values. SpatialFinder shows consistently higher agreement with ground-truth region rankings compared to baseline models. Top row: cellular diversity. Bottom row: tumor presence.

To evaluate the model’s robustness in identifying high-priority regions, we analyzed its Top-K performance by varying the value of K. Figure 5 illustrates the Top-K Overlap scores for our model and the baselines, with K varying from 10 to 1000.

Our model maintains superior performance throughout the entire range, largely surpassing the baselines at both large-scale evaluations (K = {500, 1000}) and at more focused, decision-critical levels (K = {50, 100}). Notably, in the prostate tissue, it even accurately identifies a subset of the Top-10 ground truth regions. These results highlight the model’s ability to reliably identify a compact, high-confidence set of candidate regions out of about 10,000 options, offering practical advantages for targeted downstream analyses.

### 3.3 Qualitative agreement with ground truth spatial patterns

**FIGURE 6:**
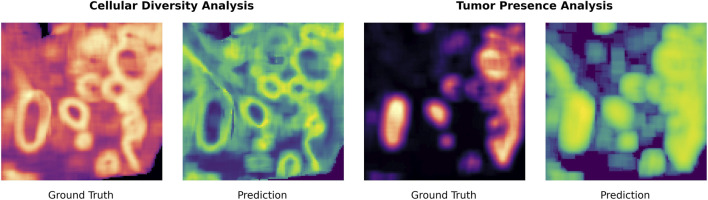
Comparison of predicted and ground-truth heatmaps for cellular diversity (left) and tumor presence (right). Each pixel corresponds to the centroid of an 8×8 μ m patch and is colored by the associated score, with brighter values indicating higher predicted or measured signal. Ground-truth maps are derived from Visium HD spatial transcriptomics, while prediction maps are generated solely from the H&E image.

In addition to quantitative metrics, we qualitatively assessed our model’s ability to capture biologically meaningful spatial patterns. Figure 6 compares heatmaps of score predictions against ground truth distributions for the prostate tissue.

The visual alignment is compelling: predicted tumor hotspots closely match high-scoring ground truth regions. The model also reliably identifies heterogeneous tissue areas, capturing the complex cellular architecture underlying transcriptomic diversity. Notably, it highlights regions at the boundary of tumor-dense zones, areas known to serve as key sites of tumor-immune interaction (Hsieh et al., 2022). Pinpointing this boundary is a central motivation in spatial transcriptomics, as these areas often represent critical sites of immune infiltration, tumor evolution, and therapeutic response (Huang et al., 2024). The model’s attention towards these biologically relevant regions underscores its ability to uncover meaningful spatial patterns beyond simple statistical correlations.

**FIGURE 7:**
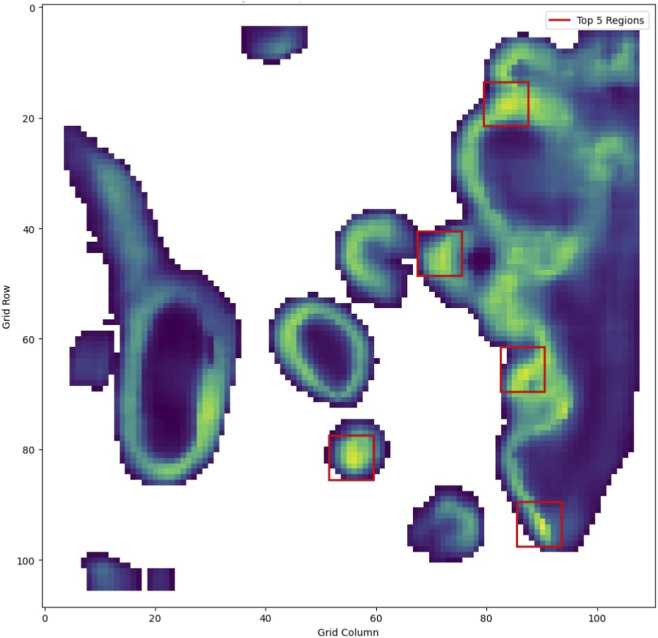
Integrated model heatmap showing the top 50% of candidate regions gated by predicted tumor presence, followed by selection based on cellular diversity. The highlighted boxes mark the top prioritized regions, which cluster along the tumor/non-tumor interface—areas typically characterized by high morphological and cellular heterogeneity.

In our experiments, we evaluated for cell diversity and tumor presence separately to assess their individual performance. However, they can also be combined or conditioned on one another to support more nuanced or multi-objective selection criteria. For instance, we also explored a two-step approach where we first applied a gating threshold on the Targeted Conditional Score to isolate tumor-enriched regions, and then ranked those regions by their Regional Diversity Score to prioritize the most heterogeneous tumor microenvironments. Figure 7 illustrates this with a heatmap of the predicted cell diversity, where each pixel represents an 8×8 patch square and its color quantifies the diversity score.

### 3.4 Synergy of cellular and visual features drives performance

**FIGURE 8:**
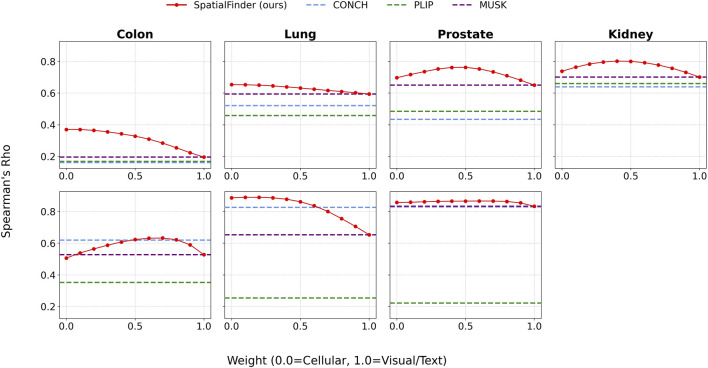
Spearman’s ρ as a function of the weighting parameter used to combine the human-in-the-loop classifier and the VLM-derived scores. Top row: cellular diversity. Bottom row: tumor presence. Across tissues, performance tends to peak at intermediate weights, producing an inverted-U pattern that indicates the hybrid combination of classifier and VLM signals outperforms either component alone.

A central premise of our approach is that integrating cellular and visual-semantic information leads to better performance than either source alone. Figure 8 illustrates this effect by plotting Spearman’s ρ as we vary the weighting parameter from 0.0 (classifier-only) to 1.0 (VLM-only) in increments of 0.2. In the majority of these cases, the highest performance is achieved with an intermediate weighting, demonstrating a meaningful synergy between modalities. The optimal weight varies by tissue and task; for practical deployment without per-tissue tuning, we recommend w=0.5 as a robust default. Even away from these optima, the blended model largely stays above other fixed-weight baselines, underscoring its robustness to imperfect weighting.

**FIGURE 9:**
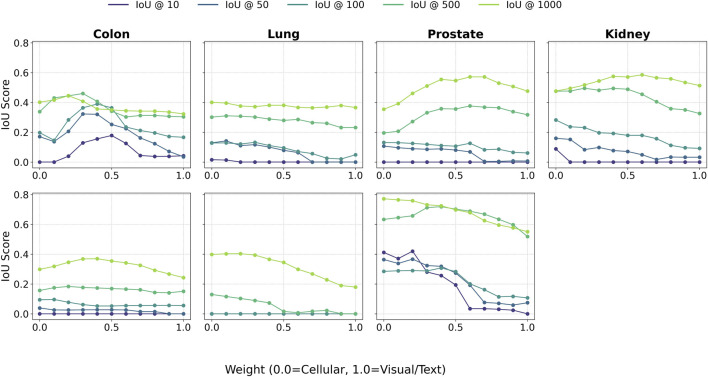
Intersection-over-Union (IoU) scores across multiple K values as a function of the weighting parameter used to combine classifier and VLM outputs. Top row: cellular diversity. Bottom row: tumor presence. Similar to the correlation analysis in Figure 8, IoU performance often peaks at intermediate weights, indicating that the hybrid combination of cellular and visual/text-based signals provides stronger alignment with ground-truth spatial rankings than either component in isolation.

This complementary effect is further illustrated in Figure 9, which shows how Top-K IoU varies with the weighting parameter. Across multiple values of K, the best performance consistently arises from a hybrid representation, reinforcing the idea that cellular and visual features offer distinct and synergistic contributions. At lower values of K, the cell classifier tends to perform better, indicating its strength in pinpointing the most salient regions.

### 3.5 Pathologist-guided tuning rapidly boosts performance

**FIGURE 10:**
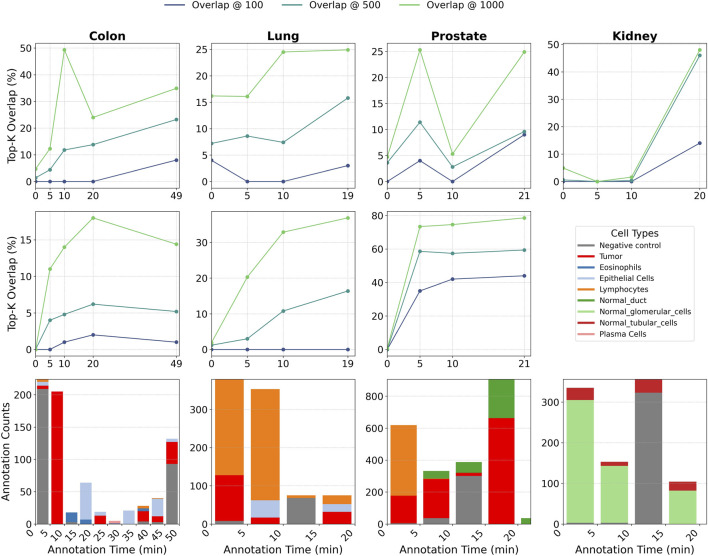
Top-K performance of SpatialFinder as a function of pathologist annotation time. Top row: cellular diversity benchmark. Middle row: tumor presence benchmark. Bottom row: number and types of cell-level annotations collected over time. As annotation time increases, overlap with ground-truth spatial rankings improves across tissues, with the largest gains occurring within the first 5–20 min of labeling. The annotation histograms illustrate how early labeling quickly supplies the model with diverse and informative cell types supporting rapid performance improvements. Beyond these early intervals, performance tends to plateau, indicating that a modest amount of targeted expert annotation is sufficient to substantially enhance the model’s region-ranking accuracy.

To evaluate the efficiency of human-in-the-loop refinement, we conducted an ablation study measuring model performance as a function of annotation time. Starting from a zero-shot classifier, we incrementally reintroduced pathologist-labeled data in timed intervals. Figure 10 displays the resulting performance trajectory on both cell diversity and tumor benchmarks.

The results show that while the zero-shot model provides a strong initialization, even minimal expert input significantly improves performance. In most tissue, as little as 5–10 min of annotation captures the majority of the total gain, particularly for broader retrieval tasks such as Top-500 or Top-1000 overlap. For example, in colon and prostate tissue, short annotations recover over 50% of the Top-1000 list, whereas kidney requires more sustained annotations (10–20 min) before a sharp performance boost is observed.

Across all benchmarks, our framework consistently outperforms the zero-shot baseline. These findings underscore its practical value: with just a few minutes of expert annotation, we can train a high-performing, tissue-specific model, enabling fast, expert-guided adaptation in real-world clinical and research environments.

Notably, performance does not increase monotonically with annotation time; transient dips are visible in several tissues (e.g., prostate diversity drops from ρ=0.56 at 5 min to ρ=0.11 at 10 min before recovering). We attribute this to three factors inherent in real-world active learning. First, pathologists often focus on 1 cell type at a time (for example, annotating primarily lymphocytes in the first 5 min, then shifting to tumor cells), which can temporarily unbalance the training set and reduce performance on metrics sensitive to cellular diversity. Second, “negative control” annotations (regions explicitly marked as background or non-informative) are necessary for classifier calibration but can transiently confuse the model when introduced in large batches; in prostate, 301 negative control cells were annotated between minutes 10–15, coinciding with the observed dip. Third, the tumor and diversity benchmarks measure different aspects of the classifier’s output: heavy annotation of tumor cells may improve tumor-targeted ranking while temporarily degrading diversity scores, and *vice versa*. These fluctuations resolve as the annotation set becomes more balanced, and the final performance consistently exceeds earlier time points across all tissues.

## 4 Discussion

We introduced SpatialFinder, a framework that combines vision-language models with pathologist-guided cell classification to identify informative subregions for spatial transcriptomics. By integrating high-level semantic features from a VLM with fine-grained cellular labels derived through active learning, our method enables targeted selection of small 500×500 μ m regions likely to capture transcriptomic heterogeneity. We demonstrate its flexibility across both discovery-driven (diversity) and hypothesis-driven (targeted) use cases, offering a practical strategy for reducing sequencing costs while preserving biological insight.

### 4.1 Limitations

A core challenge in this domain is defining what constitutes an “interesting” region (Moses and Pachter, 2022). Different use cases (e.g., tumor profiling vs. immune mapping) may demand different criteria. We addressed this by exploring both cell diversity and tumor enrichment, but these proxies remain limited. In particular, measuring diversity via our proposed methods may miss subtler or more specific compositional signals. Future work could incorporate deconvolution tools such as cell2location (Kleshchevnikov et al., 2022) or RCTD (Cable et al., 2022) to better resolve cellular heterogeneity.

The human-in-the-loop active learning component introduces several additional limitations. First, annotation variability is a concern: all annotations in this study were performed by a single board-certified pathologist, providing intra-observer consistency but no assessment of inter-observer agreement. Cell type boundaries (e.g., distinguishing “epithelial” from “tumor” cells in early-stage lesions, or “lymphocytes” from “plasma cells”) are inherently subjective, and different pathologists may apply different criteria. The “negative control” category, used to mark non-informative or ambiguous regions, is particularly prone to variability, as its definition depends on individual judgment. Second, scalability remains a practical barrier. Our workflow required 19–49 min of expert annotation per tissue, which may be acceptable for pilot studies but does not scale efficiently to large cohorts of hundreds or thousands of slides. While we showed that 5–10 min often captures the majority of performance gains, deploying this approach in clinical or high-throughput research settings would benefit from pre-trained, transferable classifiers or crowd-sourced annotation with quality control. Third, potential biases may affect the learned models. The pathologist’s annotation strategy—focusing on morphologically distinctive or clinically salient cell types first—introduces selection bias toward easily identifiable populations, potentially underrepresenting rare or ambiguous cell types. Confirmation bias may also arise if the annotator expects certain cell types in specific tissue regions and preferentially labels them. Additionally, classifiers trained on one tissue do not generalize to others without retraining; our prostate, lung, colon, and kidney models each required independent annotation sessions. Finally, our evaluation is limited to four tissue types from a single sequencing platform (Visium HD), and generalizability to other organs, disease states, patient populations, and spatial transcriptomics technologies remains to be validated.

Our evaluation relies on ranking metrics like Spearman’s ρ and Overlap@K, which are effective for global assessment but may not reflect performance in top-few selection scenarios. There is a tradeoff between optimizing overall ranking and prioritizing the highest-scoring ROIs, which could be better addressed with retrieval-style, top-K-oriented objectives that are now emphasized in visual-omics benchmarks (Shi et al., 2025; Liu, 2009). Robustness in such settings also depends on prompt design: instance-specific prompting, as in QAP, has been shown to outperform task-agnostic or pooled approaches by tailoring inputs to tissue-derived features (Yin et al., 2024).

### 4.2 Future work

Looking ahead, scaling pathology-specific vision-language models may further improve region selection in spatial transcriptomics. Models like PLIP and CONCH already show strong performance in zero-shot tissue localization tasks (Huang et al., 2023; Lu et al., 2024a), suggesting their potential for identifying biologically informative areas without retraining. Incorporating protein-level validation (e.g., imaging mass cytometry) may help confirm the molecular relevance of predicted regions (Giesen et al., 2014). As sequencing technologies vary widely in resolution and format, developing shared benchmarks will be important for comparing model outputs across platforms (You et al., 2024). In addition, iterative sequencing could make ROI selection adaptive, allowing models to refine their predictions based on early gene expression feedback (Chen et al., 2015; 2022).

The active learning workflow could also benefit from more efficient annotation strategies. Our current approach uses direct labeling, where pathologists assign absolute cell type labels; however, this can lead to non-monotonic training dynamics when annotations are unbalanced or when ambiguous cells are marked as “negative control.” Alternative paradigms such as pairwise comparisons (“Is cell A the same type as cell B?”) or relative ranking (“Which of these cells is most likely tumor?”) may reduce annotator uncertainty and yield more stable classifier updates. Active learning methods that intelligently select informative samples for annotation have already demonstrated the ability to reduce annotation effort by at least half in medical imaging applications (Zhou et al., 2021), and similar strategies tailored to cell-type labeling could further improve efficiency.

Finally, we envision adding a third reasoning layer via large language models (LLMs). Our framework leverages VLMs and classifier-driven scores; adding a generative LLM component could enable natural language queries like “tumor borders infiltrated by immune cells,” and allow the model to interpret, rank, and describe these ROIs back to the user. Pathology-specific multimodal assistants already show that coupling a vision encoder with a large language model enables natural-language question answering over slides and interactive assistance (Lu et al., 2024b).

In summary, SpatialFinder offers a step towards scalable, intelligent spatial biology. By combining VLMs with expert-in-the-loop classification, it opens a flexible, extensible foundation for guiding spatial transcriptomic experiments more efficiently and meaningfully.

## Data Availability

The original contributions presented in the study are included in the article/Supplementary Material, further inquiries can be directed to the corresponding author.
