# Supplementary material for: SpatialFinder: a human-in-the-loop vision-language framework for prioritizing high-value regions in spatial transcriptomics
Source: Front Bioinform. 2026 Apr 15;6:1746714. doi: 10.3389/fbinf.2026.1746714 (PMC13124733; doi:10.3389/fbinf.2026.1746714)
Supplement: Supplementary file 4 [file DataSheet5.pdf]

Supplementary Material

1 SUPPLEMENTARY TABLES AND SENSITIVITY ANALYSES

This supplementary document contains additional sensitivity analyses, including hyperparameter ablations and background-filter threshold robustness checks.

1.1 Supplementary Table S1: Cross-tissue UMAP dimensionality ablation

We tested UMAP dimensionalities (10, 20, 30, 40) used for computing the visual diversity component. Metrics are reported as mean $\pm$ standard deviation across tissues (colon, kidney, lung, prostate). Tumor benchmarks are reported across tumor tissues only (colon, lung, prostate).

Table S1. UMAP dimensionality ablation (cross-tissue summary).

UMAP dims	Diversity Spearman ρ	Tumor Spearman ρ
10	0.631 ± 0.186	0.783 ± 0.113
20	0.629 ± 0.184	0.783 ± 0.113
30	0.630 ± 0.185	0.783 ± 0.113
40	0.630 ± 0.186	0.783 ± 0.113

1.2 Supplementary Table S2: Stride ablation (overlap vs. computation trade-off)

We tested sliding-window strides of 1, 2, and 4 patches. Spearman ρ remains stable across strides, indicating that our choice of stride does not inflate local correlations during evaluation.

Table S2. Stride ablation (cross-tissue summary).

Stride	Diversity Spearman ρ	Tumor Spearman ρ
1	0.630 ± 0.185	0.783 ± 0.113
2	0.629 ± 0.180	0.783 ± 0.110
4	0.642 ± 0.172	0.784 ± 0.107

1.3 Supplementary Table S3: Text aggregation hyperparameter ablation (quantile)

We tested the quantile used to aggregate patch-level text-to-image similarity within a window.

Table S3. Quantile ablation (cross-tissue summary; tumor tissues only for tumor metric).

Quantile	Diversity Spearman ρ	Tumor Spearman ρ
0.70	0.630 ± 0.185	0.785 ± 0.104
0.80	0.630 ± 0.185	0.786 ± 0.109
0.90	0.630 ± 0.185	0.783 ± 0.113
0.95	0.630 ± 0.185	0.780 ± 0.117

1.4 Supplementary Table S4: Text aggregation hyperparameter ablation (exponent)

We tested the exponent used to increase contrast in the aggregated text score.

Table S4. Exponent ablation (cross-tissue summary; tumor tissues only for tumor metric).

Exponent	Diversity Spearman ρ	Tumor Spearman ρ
1	0.630 ± 0.185	0.769 ± 0.149
5	0.630 ± 0.185	0.786 ± 0.124
10	0.630 ± 0.185	0.783 ± 0.113
15	0.630 ± 0.185	0.777 ± 0.110
20	0.630 ± 0.185	0.771 ± 0.108

1.5 Supplementary Table S5: Random ROI Selection Baseline

To establish a lower bound on performance, we evaluated random ROI selection by shuffling ground truth coordinates and computing evaluation metrics. Results are averaged over 10 random seeds (mean $\pm$ std). As expected, random selection achieves Spearman's $\rho \approx 0$ (no correlation) and Overlap@10% $\approx$ 10% (the expected overlap when randomly selecting 10% of regions from a pool where 10% are ground truth). This confirms that all tested methods—including VLM-only baselines—substantially outperform chance.

Table S5. Random ROI selection baseline (10 seeds). Overlap@10% $\approx$ 10% is the expected chance level.

Tissue	Benchmark	Spearman ρ	Overlap@10%	IoU@10%
Prostate	Diversity	0.000 ± 0.000	10.0% $\pm$ 1.2%	0.359 ± 0.002
Prostate	Tumor	0.000 ± 0.000	10.0% $\pm$ 1.2%	0.236 ± 0.002
Lung	Diversity	0.000 ± 0.000	10.3% $\pm$ 1.0%	0.349 ± 0.002
Lung	Tumor	0.000 ± 0.000	10.3% $\pm$ 1.0%	0.241 ± 0.002
Colon	Diversity	0.000 ± 0.000	10.5% $\pm$ 1.1%	0.236 ± 0.001
Colon	Tumor	0.000 ± 0.000	10.5% $\pm$ 1.1%	0.293 ± 0.002
Kidney	Diversity	0.000 ± 0.000	10.0% $\pm$ 1.2%	0.412 ± 0.003

1.6 Supplementary Table S6: Background filtering threshold sensitivity (patch sampling)

The MUSK patch extraction includes background filtering to remove near-white, low-variance tiles. We evaluated robustness by sampling 300 patches per tissue and measuring the fraction flagged as background under nearby mean-intensity and standard-deviation thresholds. Results are reported as the percentage of sampled patches flagged as background.

Table S6. Background filtering sensitivity across tissues (fraction of sampled patches flagged as background).

Tissue	(mean$\geq$230, std$<$15)	(mean$\geq$235, std$<$20)	(mean$\geq$240, std$<$25)
Prostate	19.7%	19.7%	19.7%
Lung	31.3%	31.0%	31.3%
Colon	18.3%	18.3%	18.3%
Kidney	5.0%	5.0%	5.0%
